# Cross-validation of a learning climate instrument in a non-western postgraduate clinical environment

**DOI:** 10.1186/s12909-018-1127-0

**Published:** 2018-01-25

**Authors:** Jaime L. Pacifico, Cees P. M. van der Vleuten, Arno M. M. Muijtjens, Erlyn A. Sana, Sylvia Heeneman

**Affiliations:** 1 De La Salle University Medical Center, De La Salle Health Sciences Institute, 4114 Dasmarinas, Cavite Philippines; 2 Department of Educational Development and Research, Maastricht University, Maastricht, the Netherlands; 3 National Teacher Training Center for the Health Professions, University of the Philippines, Manila, Philippines; 4 Department of Pathology, Maastricht University, Maastricht, the Netherlands

**Keywords:** Educational climate, Postgraduate medical education, Learning climate, Learning climate measurement tool, Cross-cultural validation

## Abstract

**Background:**

In postgraduate training, there is a need to continuously assess the learning and working conditions to optimize learning. Students or trainees respond to the learning climate as they perceive it. The Dutch Residency Educational Climate Test (D-RECT) is a learning climate measurement tool with well-substantiated validity. However, it was originally designed for Dutch postgraduate trainees and it remains to be shown whether extrapolation to non-Western settings is viable.

The dual objective of this study was to revalidate D-RECT outside of a Western setting and to evaluate the factor structure of a recently revised version of the D-RECT containing 35 items.

**Methods:**

We invited Filipino internal medicine residents from 96 hospitals to complete the revised 35-item D-RECT. Subsequently, we performed a confirmatory factor analysis to check the fit of the 9 scale model of the revised 35-item D-RECT. Inter-rater reliability was assessed using generalizability theory.

**Results:**

Confirmatory factor analysis unveiled that the factor structure of the revised 35-item D-RECT provided a reasonable fit to the Filipino data, after removal of 7 items. Five to seven evaluations of individual residents were needed per scale to obtain a reliable result.

**Conclusion:**

Even in a non-Western setting, the D-RECT exhibited psychometric validity. This study validated the factor structure of the revised 35-item D-RECT after some modifications. We recommend that its application be extended to other Asian countries and specialties.

## Background

It is recognized that the way students or trainees respond to education is conditioned by the way they perceive their educational climate. “Educational climate”, in this sense, denotes a manifestation of the educational environment and of the curriculum [1] and is often used interchangeably with the concept “educational environment” in many educational researches [2]. In postgraduate medical training, it has been demonstrated that a learning climate can be optimized by integrating work and training in a way that is keyed to the particular needs of the trainees [3]. Learning instruments have been designed to measure learning climates in postgraduate training, such as the Postgraduate Hospital Educational Environment Measure (PHEEM) [4] and Dutch Residency Educational Climate Test (D-RECT) [5]. The Dundee Ready Education Environment Measure (DREEM) [6] although originally designed for undergraduate medical students, has also been used in postgraduate training [7].

What is more, recent years have seen a proliferation of detailed reviews of the learning environment tools [2, 8, 9]. Colbert-Getz et al. [8], for instance, evaluated the evidence substantiating the validity of 28 learning environment tools, 13 of which were used in postgraduate training. It transpired that the validity of only 3 of these 13 instruments was sufficiently substantiated, D-RECT being one of these three. Peculiarly, however, the authors observed that, even though the validity of D-RECT was much better supported, DREEM enjoyed wider currency. Their study had been inspired, in part, by the incentive to encourage educators and researchers to choose learning environment tools based on good validity evidence. From a different angle, Schönrock-Adema et al. [9], who looked into the theoretical framework of existing learning instruments, observed that D-RECT had a strong theoretical foundation, particularly in relation to socio-cultural concepts compared with other learning environment instruments.

The D-RECT [5], indeed, is a product of sound theoretical data from previous research combined with expert opinion then subjected to factor and generalizability analysis. It has been developed following the recommended steps in constructing a good instrument [10]. The rigor with which D-RECT has been developed and the reviews above indicate that D-RECT, as one of the current learning environment tools, has the attributes of a good instrument. Though these findings sound promising, we do not yet know whether they can be extrapolated to countries beyond the Netherlands, as the D-RECT was originally designed for Dutch postgraduate medical trainees [5]. In a study of problem-based learning (PBL) and self-directed learning (SDL) across three culturally diverse regions, Frambach, Driessen, Chan & van der Vleuten [11] identified specific cultural and contextual factors that initially inhibited the applicability of PBL and SDL across different cultures. This begs the question of whether cultural differences can act as a barrier to the effectiveness of a learning climate measurement instrument.

For these reasons we decided to investigate the applicability of D-RECT outside of the Netherlands. The D-RECT has recently been revisited by Silkens, Smirnova, Stalmeijer, Arah, Scherpbier, van der Vleuten & Lombarts [12] who proposed a new, 9-factor structure with a 35-item questionnaire, in contrast to the original, 50-item test. As with the first study, this second study was conducted in the Netherlands. In summary, the objective of the current study was to revalidate the revised 35-item D-RECT in a non-Western context; we investigated its internal structure and internal consistency during use in an Asian setting to measure the learning climate in residency training. Lastly, we conducted a generalizability study to evaluate what minimum number of respondents would be needed to obtain a reliable result.

## Methods

### Setting

We conducted this study in the Philippines and respondents were all second- or third-year internal medicine residents from 96 hospitals with accredited internal medicine residency training programs. The questionnaires were in English which is the language of instruction in the Philippines from primary to tertiary education. In October of the second and third year of residency, these trainees take a compulsory annual multiple-choice question test as part of an in-training examination. At the end of the examination, and with the approval of the Philippine College of Physicians (PCP), which regulates internal medicine residency programs in the country, we invited around 1000 trainees to complete the D-RECT questionnaire, on a purely voluntary basis.

The typical residency programs in internal medicine in the Philippines has a minimum of two residents per year level for the smaller hospitals and as much as twenty residents per year level in the big government hospitals. First year residents are usually assigned at the wards, second year residents rotate across emergency rooms and third year residents man the intensive care units, respond to the inter-departmental referrals and support the first year residents in their duties at the ward. A resident is required to conduct two research projects during the 3 years of residency. The residents also are assigned patients at the out patient department. There are regular conferences such as mortality and morbidity conferences, emergency room case-conferences, grand rounds and endorsement conferences where residents are required to present. Once a year all the residents take the residents in training examination (RITE), a multiple choice question type of written examination conducted at the national level. At the end of each year residents are evaluated whether they will be promoted to the next year level or graduate, by the residency training committee. The basis of the annual evaluations and promotions are written examinations, OSCEs, compliance with research, attitude and clinical competence which are evaluated using a Likert scale questionnaire, filled out by consultants of the department.

In the Philippines, entry into a medical school requires a college degree similar to that of the United States. Before medical graduates can practice medicine or pursue further training, they have to take a licensure examination. The internal medicine residency program lasts three years and is a prerequisite to subspecialty training (cardiology, for example). Unlike the Netherlands, the Philippines has a hierarchical culture: as in other Asian cultures, the teacher is considered as knowledge expert [13]. This is true of any educational setting and in a postgraduate training program this can have a ‘hidden curriculum’ type of effect such that performance and evaluation of trainees are impacted more by personalities rather than by policies.

### Data analysis

As the D-RECT involves data referring to the evaluation of the educational climate, the department was used as the unit of analysis. As previously stated, we recruited our respondents from 96 different internal medicine hospital departments. Hence, before starting the analysis, we aggregated residents’ responses to the level of the department, computed the mean scores across residents for each department, and used these in the factor analysis. Although factor analysis is ideally performed with a subject-to-item ratio of 10:1, which, in our case, would translate into a minimum of 500 units, we take it that this requirement has been met as aggregate departmental scores were based on responses from 843 residents. Since some responses were not accepted, the analysis, ultimately, was based on aggregates of 93 (out of the 96) hospital departments. The final result of the analysis is a mean reflecting responses by hundreds of residents, lending stability to the factor structure estimation.

We performed a confirmatory factor analysis (CFA) to determine if the residents’ aggregate data fitted the 9-factor structure of the revised 35-item D-RECT [12]. We used the following criteria and associated pre-determined cut-off values to gauge goodness of fit: relative Chi-square (CMIN/DF < 2), goodness-of-fit index (GFI > 0.9), Tucker-Lewis index (TLI > 0.9), comparative fit index (CFI > 0.9), and the root mean square error of approximation (RMSEA < 0.08). In the present study a sufficient fit was deemed to have been achieved when 4 out of 5 criteria had been met with significant results. We checked the internal consistency of the scales by obtaining the Cronbach’s alpha for each of the scales. We used Amos structural equation modeling software for the confirmatory factor analysis.

In the generalizability study that was subsequently conducted, we estimated the criterion for the standard error of measurement (SEM) to be 0.26 (1.96 × 0.26 × 2 = 1), hence assuming a maximum “noise level” of 1, as in the study by Boor et al. [5]*.* The SEM refers to the standard deviation of scores that would have been obtained in a single trainee, in the condition that the trainee had been tested multiple times. In contrast, the standard deviation (SD) refers to scores obtained by a group of trainees on a single test [14]. The SEM can be interpreted on the original scoring scale and is useful to set a maximum level of acceptable “noise”. With an SEM of 0.26 we can reliably differentiate 1 point on the 5-point Likert-type scale.

Finally, for each scale of the 28-item version of the revised 35-item D-RECT we performed a generalizability analysis for the respondent-level scores to determine the number of residents needed to achieve a reliable score.

## Results

In the analysis we excluded questionnaires that had 10 or more unanswered items (8), contained no institutional reference (48), presented all or all but one with identical answers (101), or came from hospital departments with only 2 or less respondents (6). Of the original 1006 questionnaires evaluating 96 departments, 843 (84%) evaluating 93 departments remained to enter the analysis. The next step was to apply CFA to the department-level data using the 9 scales model of the revised 35-item D-RECT [12]. Initially, the revised D-RECT did not provide sufficient fit. We decided to investigate whether such a fit could be obtained upon removal of certain items. In order to be guided which items needed to be removed, the modification index (MI) was used as an indicator. The value of this index represents the expected drop in overall Chi-square value when the parameter is to be freely estimated. Thus the MI indicates the potential gain of fit when removing an item from the model. By subsequently removing 7 items with a high MI, we obtained a 9 scale model with a satisfactory fit fulfilling 4 out of the 5 fit criteria. Upon further analysis, there were no specific statistical reasons e.g. exceptional MI, SD, skewness or kurtosis, why these seven items had to be removed, other than their negative contribution to the model fit. The improvement of fit resulting from removal of an item from its scale indicated that the item’s score was not consistent with the scale score. This resulted in a 28-item questionnaire with the same factor structure as in the study by Silkens et al. [12]. Table 1 presents the descriptive statistics of the score for each of the scales. The lowest score was 2 in the scales peer collaboration and accessibility of supervisors. The highest was 5 in the scales teamwork, formal education and patient sign-out. The between-department standard deviation (SD) of each of the 9 scale scores was relatively low, ranging from 0.28 to 0.37. In the original D-RECT study [5] the standard deviation was 0.69 to 1.27, while in the last study which revisited the D-RECT tool, which was done also at the Netherlands, the SD was 0.59 to 0.81 [12].

**Table 1:** Descriptive Statistics of the 9 scales of the 28-item Revised D-RECT for the 93 departments

	Minimum	Maximum	Mean	Std. Deviation
Coaching and assessment	2.16	4.80	3.92	0.32
Teamwork	2.47	5.00	4.04	0.31
Peer Collaboration	2.00	4.89	4.09	0.37
Educational atmosphere	2.20	4.50	3.85	0.29
Work is adapted to resident’s competence	2.33	4.78	4.06	0.28
Accessibility of supervisors	2.00	5.00	4.14	0.33
Formal education	2.47	5.00	4.06	0.28
Role of specialty tutor	2.27	4.78	3.83	0.32
Patient sign out	2.20	5.00	4.03	0.30

As mentioned, the inference of a reasonable fit was based on the fulfillment of 4 out of 5 of the following criteria: CMIN/DF, GFI, TLI, CFI, and RMSEA (Table 2). Additionally, for all the 9 scales we found high Cronbach’s alpha coefficients, ranging from 0.85 to 0.96. This confirmed that all the 9 scales of the 28-item D-RECT, which was based on the revised 35-item version by Silkens et al. [12], had high internal consistency (Table 3). Generalizability analysis showed that for the scales coaching and assessment, educational atmosphere, work is adapted to resident’s competence accessibility, formal education and patient sign-out, 5 respondents were needed for a reliable outcome. For the scales teamwork, peer collaboration and accessibility of supervisors a minimum of 6 respondents can give a reliable result. For the scale specialty tutor 7 respondents were needed (Table 4).

**Table 2:** Fit indices for the 9-factor structure of revised 35-item D-RECT and for the 28-item version

Criteria	35-item revised D-RECT	28-item revised D-RECT^a^
CMIN/DF < 2.00	2.066	1.573
GFI > 0.90	0.620	0.746
TLI > 0.90	0.825	0.922
CFI > 0.90	0.846	0.936
RMSEA < 0.08	0.108	0.079

^a^Excluded items, see Table 5

**Table 3:** Reliability between items (Cronbach’s alpha) for each scale of the 28-item revised D-RECT

Scales	Item number	Cronbach’s alpha
Coaching and assessment	1, 2, 3, 4, 5	0.92
Teamwork	6, 7, 8	0.93
Peer collaboration	9, 10, 11	0.92
Educational atmosphere	12, 13, 14, 15	0.85
Work is adapted to resident’s competence	16, 17, 18	0.88
Accessibility of supervisors	19, 20	0.93
Formal education	21, 22, 23	0.88
Role of specialty tutor	24, 25, 26	0.96
Patient sign out	27, 28	0.91

**Table 4:** Standard error of measurement (SEM) and sample size

*N Respondents*	1	2	3	4	5	6	7	8	9
Scales	Standard Error of Measurement								
Coaching and assessment	0.56	0.39	0.32	0.28	**0.25**	0.23	0.21	0.20	0.19
Teamwork	0.57	0.40	0.33	0.29	0.26	**0.23**	0.22	0.20	0.19
Peer Collaboration	0.60	0.42	0.35	0.30	0.27	**0.24**	0.23	0.21	0.20
Educational Atmosphere	0.54	0.38	0.31	0.27	**0.24**	0.22	0.20	0.19	0.18
Work is adapted to resident’s competence	0.52	0.36	0.30	0.26	**0.23**	0.21	0.20	0.18	0.17
Accessibility of supervisors	0.58	0.41	0.34	0.29	0.26	**0.24**	0.22	0.21	0.19
Formal Education	0.52	0.37	0.30	0.26	**0.23**	0.21	0.20	0.19	0.17
Role of Specialty tutor	0.67	0.47	0.39	0.33	0.30	0.27	**0.25**	0.24	0.22
Patient Sign-out	0.57	0.40	0.33	0.28	**0.25**	0.23	0.21	0.20	0.19

Table 5 shows the final 28-item revised D-RECT. Shown in italics are the 7 items which are part of the 35-item revised D-RECT but were removed to achieve a fit with our data.

**Table 5:** Items and scales of the 35-item revised D-RECT and the 28-item version that was shown to fit the Filipino internal medicine data

Coaching and Assessment
1. My attendings take the initiative to evaluate my performance
2. My attendings take the initiative to evaluate difficult situations I have been involved in
3. My attendings evaluate whether my performance in patient care is commensurate with my level of training
4. My attendings occasionally observe me taking a history
5. My attendings give regular feedback on my strengths and weaknesses
* [My attendings assess not only my medical expertise but also other skills such as teamwork, organization or professional behavior]* ^a^
Teamwork
6. Attendings, nursing staff, other allied health professionals and residents work together as a team
7. Nursing staff and other allied health professionals make a positive contribution to my training
8. Nursing staff and other allied health professionals are willing to reflect with me on the delivery of patient care
Peer collaboration
9. Residents work well together
10. Residents, as a group, make sure the day’s work gets done
11. With our group of residents it is easy to find someone to cover or exchange a call
Educational atmosphere
12. Continuity of care is not affected by differences of opinion between attendings
13. Differences of opinion between attendings about patient management are discussed in such a manner that is instructive to others present
14. There is (are) NO attending physician(s) who have a negative impact on the educational climate
15. My attendings treat me with respect
* [Differences of opinion are not such that they have a negative impact on the work climate]* ^a^
Work is adapted to resident’s competence
16. The work I am doing is commensurate with my level of experience
17. The work I am doing suits my learning objectives at this stage of my training
18. It is possible to do follow up with patients
Accessibility of supervisors
19. When I need an attending, I can always contact one
20. When I need to consult an attending, they are readily available
* [It is clear which attending supervises me]* ^a^
Formal education
21. Residents are generally able to attend scheduled educational activities
22. Attendings contribute actively to the delivery of high quality formal education
23. Formal education and training activities are appropriate to my needs
* [Educational activities take place as scheduled]* ^a^
Role of Specialty Tutor
24. The specialty tutor monitors the progress of my training
25. The specialty tutor provides guidance to other attendings when needed
26. The specialty tutor is actively involved in improving the quality of education and training
* [In this rotation evaluations are useful discussions about my performance]* ^a^
* [My plans for the future are part of the discussion]* ^a^
* [During evaluations inputs from several attendings is considered]* ^a^
Patient sign out
27. Sign out is used as a teaching opportunity
28. Attendings encourage residents to join in the discussion during sign out

^a^Item of the 35-item revised D-RECT that was not included in the 28-item version

## Discussion

With this cross-validation study we have demonstrated that the D-RECT, although originally designed for postgraduate medical trainees in the Netherlands, is useful in a non-Western setting. Moreover, we validated the internal consistency and internal 9-factor structure of the revised D-RECT [12].

Our validation study delivered a 28 item instrument which we have shown can be used in a setting with a significantly different culture from the original resident trainees it was designed for. The Netherlands has an open society in contrast with the hierarchical culture in the Philippines which is similar to many southeast Asian countries. It probably is the strength of the D-RECT instrument having undergone a rigid methodical process that makes it useful outside the Netherland as a learning climate tool in spite of the potential cultural differences that exist with regards post-graduate medical training [15] and non-medical higher education [13] between the Netherlands and the Philippines.

Another good quality of the D-RECT is that it contains many items representing sociocultural aspects of learning [9]. Sociocultural theory proposes that learning sets in when residents participate in the daily activities of patient care, interacting with peers and senior members of the health team and allied health professionals, also referred to as their “community of practice” [16–18]. One illustrative example in the D-RECT is the “patient sign out” scale which refers to all interpersonal actions required to achieve a seamless transition of care from one resident to another. This seemingly routine and trivial activity is one instance where, consistent with sociocultural theory, interactions with peers can stimulate learning. Teunissen et al. [19] have actually shown that participation by trainees in work-based activities constitutes the first step that initiates the process of learning.

In our Philippine setting, analysis indicated that we could retain the 9-factor structure of the revised D-RECT, however for a proper fit to the model, we could use only 28 of the 35 items of the revised instrument [12]. The obvious advantage of a shorter instrument is its increased efficiency, consistent with the recommendation that learning instruments should be easy to complete, as this encourages respondents to provide more truthful answers [8]. The generalizability study revealed that 5–6 evaluations were needed per scale for a reliable inference of 1 point on the scoring scale, the “specialty tutor” scale, which required 7 evaluations, excepted. In the revised 35-item D-RECT study, by contrast, a minimum of 8 residents were required to arrive at reliable outcome [12].

Application of D-RECT will provide training officers with valuable feedback regarding the learning climate, allowing them to monitor their training program, institute and implement changes as needed [5]. The questionnaire, then, becomes an instrument by which trainees can voice what they feel are weak or lacking in the training program without disclosing their identity and fearing possible consequences from the administrators. Administrators, in turn, will also be made aware of the institution’s successes. As such, we would welcome a wider use of tools that measure the learning environment like the D-RECT, as these can play a pivotal role in monitoring the quality of health care. Just as the United States has launched initiatives to review the clinical learning environment, likewise a tool like D-RECT can help in collecting information to improve institutional participation and graduate medical education [20].

In contemplating possible strengths and weaknesses of the present research, we believe the fact that all the country’s hospitals offering internal medicine residency programs were represented can be considered an asset. Conversely, our narrow focus on a single specialty, that of internal medicine may explain the low standard deviation noted between departments.

This creates an impetus for future studies which target multiple specialties in order to investigate whether our findings can be extrapolated to pediatric settings and residency training programs with surgical skills like surgery and obstetrics-gynecology. A similar evaluation among Asians based on a larger and more diverse sample could further bolster the applicability of D-RECT outside of a Western setting.

## Conclusion

When used for the evaluation of the clinical learning environment in a non-Western postgraduate training setting, the D-RECT, in its revised form, exhibited psychometric validity. We recommend that its application be extended to other Asian clinical training programs and specialties.

## Note

The abstract of this paper was published in the AMEE 2016 proceedings [21]**.**

## Abbreviations

CFA: Confirmatory Factor Analysis
CFI: Comparative-fit index
CMIN/DF: Relative Chi-square
D-RECT: Dutch Residency Educational Climate Test
GFI: Goodness-of-fit index
MI: Modification Index
RMSEA: Root mean square error of approximation
SD: Standard deviation
SEM: Standard error of measurement
TLI: Tucker-Lewis index
